# MICU3 regulates mitochondrial Ca^2+^-dependent antioxidant response in skeletal muscle aging

**DOI:** 10.1038/s41419-021-04400-5

**Published:** 2021-11-29

**Authors:** Yun-Fei Yang, Wu Yang, Zhi-Yin Liao, Yong-Xin Wu, Zhen Fan, Ai Guo, Jing Yu, Qiu-Nan Chen, Jiang-Hao Wu, Jing Zhou, Qian Xiao

**Affiliations:** 1 Department of Geriatrics, The First Affiliated Hospital of Chongqing Medical University, Chongqing, China; 2 Department of Orthopedics, The First Affiliated Hospital of Chongqing Medical University, Chongqing, China; 3 Department of Geriatrics, Sichuan Academy of Medical Science & Sichuan Province People’s Hospital, Chengdu, Sichuan China; 4 Department of Clinic, Chongqing Medical and Pharmaceutical College, Chongqing, China

**Keywords:** Ageing, Calcium and phosphate metabolic disorders

## Abstract

Age-related loss of skeletal muscle mass and function, termed sarcopenia, could impair the quality of life in the elderly. The mechanisms involved in skeletal muscle aging are intricate and largely unknown. However, more and more evidence demonstrated that mitochondrial dysfunction and apoptosis also play an important role in skeletal muscle aging. Recent studies have shown that mitochondrial calcium uniporter (MCU)-mediated mitochondrial calcium affects skeletal muscle mass and function by affecting mitochondrial function. During aging, we observed downregulated expression of mitochondrial calcium uptake family member3 (MICU3) in skeletal muscle, a regulator of MCU, which resulted in a significant reduction in mitochondrial calcium uptake. However, the role of MICU3 in skeletal muscle aging remains poorly understood. Therefore, we investigated the effect of MICU3 on the skeletal muscle of aged mice and senescent C2C12 cells induced by d-gal. Downregulation of MICU3 was associated with decreased myogenesis but increased oxidative stress and apoptosis. Reconstitution of MICU3 enhanced antioxidants, prevented the accumulation of mitochondrial ROS, decreased apoptosis, and increased myogenesis. These findings indicate that MICU3 might promote mitochondrial Ca^2+^ homeostasis and function, attenuate oxidative stress and apoptosis, and restore skeletal muscle mass and function. Therefore, MICU3 may be a potential therapeutic target in skeletal muscle aging.

## Introduction

Skeletal muscle plays an essential role in metabolic health and physical function [1]. Loss of skeletal muscle mass and function is an important manifestation of aging. Age-related skeletal muscle disorder involving the accelerated loss of muscle mass and function is associated with falls, frailty, and mortality [2, 3]. Studies have shown that insulin resistance, inflammation, denervation, and autophagy can cause skeletal muscle disorder [4–6]. However, the mechanism underlying skeletal muscle aging is still not fully elucidated.

Growing evidence has suggested that mitochondrial dysfunction may play a critical role in the pathogenesis of skeletal muscle aging [7–9]. For example, one study has demonstrated that mitochondria isolated from skeletal muscles of aging mice exhibit bioenergetic defects [10]. Recent data showed that mitochondrial respiratory function defects, oxidative stress, mitochondrial DNA (mtDNA) damage, and metabolic disturbance might impact aging skeletal muscle [11, 12]. Therefore, it is important to protect mitochondrial health during aging [12]. However, despite increasing data implicating mitochondrial pathology in skeletal muscle aging, the mechanisms underlying these processes remain largely unknown.

Mitochondrial Ca^2+^ uptake is essential for the regulation of the intrinsic function of mitochondria. One of the most important functions of mitochondrial Ca^2+^ is the regulation of metabolic activity [13]. One study has reported that dysregulation of intramitochondrial calcium results in impaired mitochondrial calcium uniporter complex (MCUC) function and abnormal mitochondrial metabolism and dynamics [14]. Mitochondrial Ca^2+^ also plays a role in oxidative stress. It was reported that neuromuscular junctions oxidative stress resulted in muscle mitochondria calcium handling defects in mice deficient in Cu, Zn-superoxide dismutase [15]. Ca^2+^-associated cell death is often mediated by mitochondria, as mitochondrial Ca^2+^ overload triggers the opening of the mitochondrial permeability transition pore (mPTP) and subsequent cell death [16]. One critical aspect of mitochondrial Ca^2+^ accumulation is its sigmoidal response to cytoplasmic Ca^2+^ levels [13]. In photoreceptors, the precise localization of mitochondria to the ellipsoid protected the cell body from the cytosolic Ca^2+^ that accumulated in the outer segment in darkness, and uptake of Ca^2+^ into mitochondria also influenced their energetic output [17]. Interestingly, it has been reported that reduced skeletal muscle mitochondrial calcium uptake during the stimulation process was observed in fatigued patients and mice [18–21]. However, whether mitochondrial calcium handing is involved in skeletal muscle aging remains to be defined.

In recent years, the mitochondrial calcium uniporter (MCU) and its regulatory subunits MICU1 (mitochondrial calcium uptake 1) and MICU2 (mitochondrial calcium uptake 2) have become hot topics in mitochondrial research. Ca^2+^ import into the mitochondrial matrix occurs via the mitochondrial Ca^2+^ uniporter complex (MCU), which is comprised of a multimer of the pore-forming protein MCU and many associated regulatory proteins [22–25]. Previous studies reported that MCU and MICU1 mRNA levels were downregulated in fatigued patients, and knockdown of MCU or MICU1 led to skeletal muscle atrophy or weakness [21, 26, 27]. In addition, MICU3 has been discovered as a new MCU regulator in the nervous system, which could increase mitochondrial Ca^2+^ uptake by working with MICU1 [28]. The content of MICU3 in skeletal muscle is second only to the nervous system, but its function in skeletal muscle, to our knowledge, has never been reported.

In this study, we identified that MICU3 was downregulated in the aged mice skeletal muscle and in senescent C2C12 cells induced by d-gal. And the downregulated MICU3 was a contributing factor to the oxidative stress production and apoptotic process during aging. Moreover, overexpression of MICU3 effectively alleviated the loss of skeletal muscle mass and function via promoting mitochondrial Ca^2+^ homeostasis to inhibit ROS-mediated apoptosis. These findings suggest that upregulating MICU3 expression may be a potential therapeutic strategy in skeletal muscle disorders associated with aging, such as sarcopenia.

## Results

### MICU3 was downregulated in skeletal muscle from aged mice and senescent C2C12 cells induced by d-gal

**Fig. 1:**
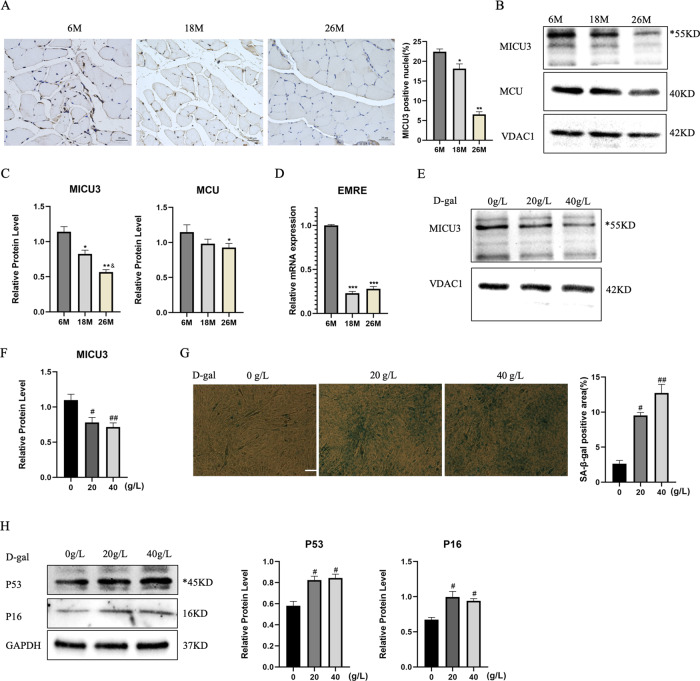
MICU3 was downregulated in skeletal muscle from old mice and senescent C2C12 cells induced by d-gal. **A** IHC for MICU3 in mice gastrocnemius muscle (magnification ×400; scale bar = 20 μm). **B**, **C** Western blot and quantification of MICU3 and MCU protein level in mice gastrocnemius muscle. VDAC was used as the loading control. *indicates target bands. **D** The mRNA level of EMRE in mice gastrocnemius muscle. **E**, **F** Western blot and quantification for MICU3 in C2C12 cells. VDAC was used as the loading control. *indicates target bands. **G** SA-*β*-gal staining and quantification for C2C12 cells (scale bar = 50 μm). **H** Western blot and quantification for P16 and P53 in C2C12 cells. *indicates target bands. GAPDH was used as the loading control. Data were expressed as mean ± SEM, and data were analyzed using a one-way ANOVA. *p < 0.05 vs. the 6 M group; ^&^*p* < 0.05, vs. The 18M group; *n* = 3 mice in each group; ^#^*p* < 0.05, vs. the 0 g/L group; *n* = 3.

To investigate the alterations in MICU3 in skeletal muscle of aging mice, western blotting and IHC were used. The IHC and western blot results showed that the expression of MICU3 was decreased in skeletal muscle from 18- to 26-months-old mice, accompanied by the decrease in the protein level of the uniporter MCU. Due to the lack of antibodies for the regulator EMRE, we detected the mRNA level of EMRE, which also declined during aging [Fig. 1A–D].

Previously, our team found that d-gal could induce C2C12 cell senescence [29], so we used d-gal to generate a senescent cell model. In this model, we found that MICU3 was decreased [Fig. 1E, F]. SA-*β*-gal staining was performed to investigate the effect of d-gal on C2C12 cell senescence [Fig. 1G]. The results demonstrated that the SA-*β*-gal-positive cells were increased after d-gal treatment. We also found that the myogenic markers (MyoD and myogenin) were decreased in senescent C2C12 cells exposed to d-gal concentrations of 20 and 40 g/L with an increase of the senescent markers (P16 and P53) [30] [Fig. 1H]. These results indicate that pre-treatment with d-gal promoted pro-senescence effects on C2C12 cells and decreased the expression of MICU3.

### Downregulation of MICU3 in C2C12 cells impaired the differentiation capacity and induced mitochondrial dysfunction

**Fig. 2:**
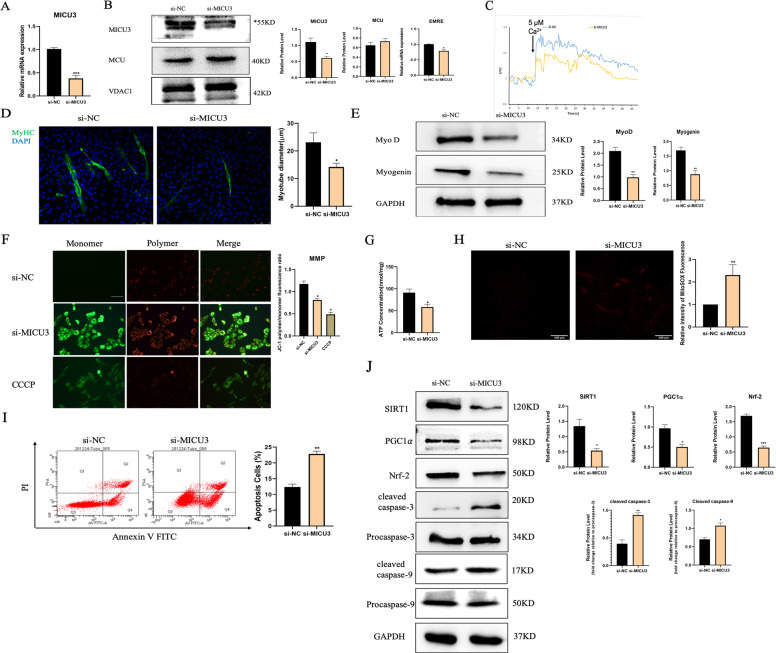
Downregulation of MICU3 in C2C12 cells induced impairment of differentiation capacity and mitochondrial dysfunction. **A** The mRNA level of MICU3 in C2C12 cells. **B** Western blot and quantification for MICU3 and MCU, and mRNA level of EMRE. VDAC was used as the loading control for western blot, and GAPDH was used as the loading control for RT-PCR. *indicates target bands. **C** The mitochondrial calcium uptake assay in C2C12 cells. *F*_0_ = Initial fluorescence value, *F* = Arbitrary fluorescence values. **D** The photo and quantification of myotubes (scale bar = 75 μm). **E** Western blot and quantification for MyoD, and Myogenin. GAPDH was used as the loading control. **F** The fluorescence staining for JC-1, indicator of mitochondrial membrane potential (scale bar = 100 μm). CCCP was used as a positive control. **G** The ATP concentration of C2C12 cells. **H** The fluorescence staining for MitoSOX, indicator of mitochondrial ROS (scale bar = 100 μm). **I** Representative pictures of flow cytometry analysis in Annexin V- FITC/PI staining. (J)Western blot and quantification of SIRT1, PGC1*α*, Nrf-2, cleaved caspase-3, cleaved caspase-9, Procaspase-3, and Procaspase-9. GAPDH was used as the loading control. Data were expressed as means ± SEM, and data were analyzed using *t*-test and one-way ANOVA. *p < 0.05 vs. the si-NC group; *n* = 3.

Our PCR and western blot data showed that MICU3 siRNA significantly reduced the expression of MICU3 and EMRE without affecting the MCU expression [Fig. 2A, B]. The size of the myotubes in the si-MICU3 group was smaller than in the si-NC group [Fig. 2D]. We also found that knockdown of MICU3 decreased the expression of MyoD and myogenin [Fig. 2E], which confirms that downregulation of MICU3 impaired the differentiation capacity of C2C12 cells.

The mitochondrial Ca^2+^ uptake capacity was markedly reduced in the C2C12 cells due to MICU3 knockdown [Fig. 2C]. Furthermore, we found that the production of ATP was significantly decreased by MICU3 knockdown [Fig. 2G]. As shown in Fig. 2F, the knockdown of MICU3 was characterized by a slightly higher JC-1 monomer fluorescence and a significantly reduced JC-1 polymer fluorescence. The ratio of polymer-to-monomer fluorescence was decreased in the si-MICU3 group, demonstrating that the knockdown of MICU3 reduced the mitochondrial membrane potential.

The production of mitoROS and apoptotic cells increased significantly by MICU3 deficiency [Fig. 2H, I]. The western blot results also showed that the expression of the oxidative stress-related pathway SIRT1/PGC1*α*/Nrf-2 was decreased in the si-MICU3 group while the expression of cleaved caspase-3 and cleaved caspase-9 was increased [31] [Fig. 2J]. These results indicate that knockdown of MICU3 could induce mitochondrial disorder and apoptosis.

### Overexpression of MICU3 improved muscle mass and function in aging mice

**Fig. 3:**
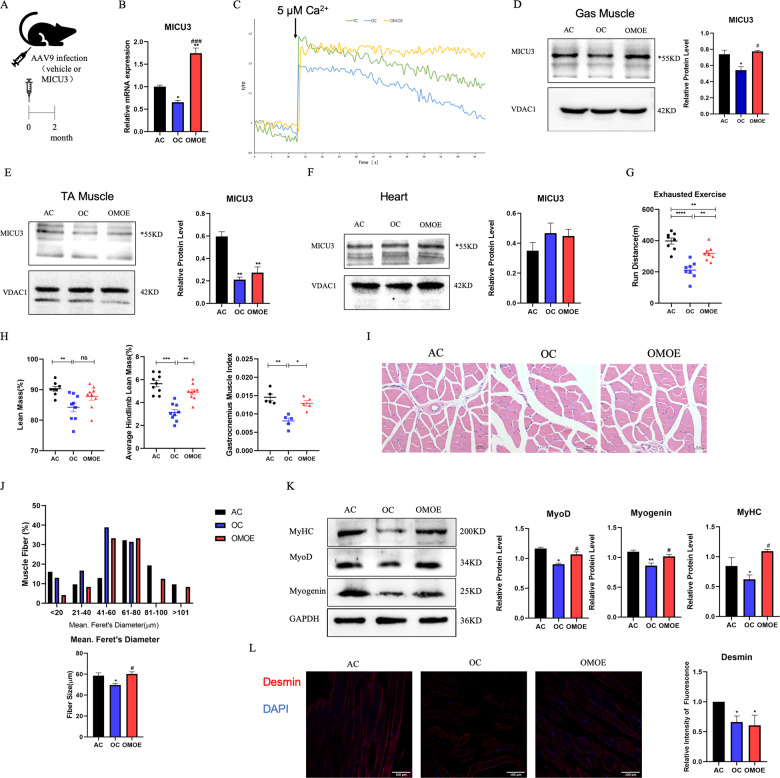
Overexpression of MICU3 improved aging mice muscle mass and function. **A** Schematic diagram of AAV9 injection. **B** The mRNA level of MICU3 in mice gastrocnemius muscle. **C** The mitochondrial calcium uptake assay in primary myoblasts from mice gastrocnemius muscle. **D**–**F** Western blot and quantification for MICU3 in mice gastrocnemius muscle, tibialis anterior (TA) muscle, and myocardium. VDAC was used as the loading control. *indicates target bands. **G** The furthest running distance in exhausted exercise. **H** Lean mass (%), defined as lean mass/body weight. Average hindlimb lean mass (%), defined as average hindlimb lean mass/body weight. Gastrocnemius muscle index (GMI), defined as the gastrocnemius wet weight/body weight. **I**, **J** Myofibers were stained with H&E (magnification ×400; scale bar = 20 μm), and the Feret’s diameter of the gastrocnemius muscle fibers was measured by ImageProPlus software. **K** Western blot for MyoD, Myogenin and MyHC. GAPDH was used as the loading control. **L** The immunofluorescence of Desmin in mice gastrocnemius muscle. (magnification = ×200; scale bar = 100 μm). Data were expressed as means ± SEM, and data were analyzed using a one-way ANOVA. *p < 0.05 vs. the AC group; ^#^*p* < 0.05 vs. the OC group; *n* = 3 mice per group for western blot and RT-PCR analyses; *n* = 4 for H&E staining; *n* = 5 for GMI measurements; *n* = 9 for dual-energy X-ray absorptiometry (DEXA) measurements.

We further attempted to determine whether the overexpression of MICU3 would protect skeletal muscle against age. An intramuscular injection of AAV9 encoding MICU3 was given to aged mice [Fig. 3A]. According to the RT-PCR and western blot data, injection of MICU3-overexpression AAV9 resulted in an increase in gastrocnemius muscle MICU3 expression compared to controls [Fig. 3B, D]. However, injection of the virus into the gastrocnemius muscle did not increase the expression of MICU3 in the tibial anterior muscle (TA) or myocardium [Fig. 3E, F], indicating that in situ injection of the virus may have little effect on other tissues. Moreover, the exhausted exercise results demonstrated that, compared to the AC group, aging decreased the skeletal muscle function in the OC group, but overexpression of MICU3 in aged mice increased the skeletal muscle function [Fig. 3G]. The Dual-energy X-ray absorptiometry (DEXA) results exhibited that the lean mass of the old control (OC, 26M) group was lower than the adult control (AC, 6M) group, but the lean mass of the old MICU3-overexpression (OMOE, 26M) group did not increase significantly [Fig. 3H]. Compared to the AC group, the average hindlimb lean mass and gastrocnemius muscle index (GMI) of the OC group were obviously lower. However, overexpression of MICU3 in aging mice increased these two indicators [Fig. 3H]. In addition, the muscle fiber Feret’s diameter was reduced in the OC group compared to the AC group but were increased in the OMOE group [Fig. 3I, J]. As shown in Fig. 3K, the myogenic markers MyoD, Myogenin, and MyHC were lower in the OC group but were increased by the overexpression of MICU3. By staining with Desmin, a satellite cell marker, we found that aging decreased the number of satellite cells, and overexpression of MICU3 did not improve it [Fig. 3L], suggesting that MICU3 may alleviate skeletal muscle mass by increasing the differential capacity, not number, of satellite cells.

### Overexpression of MICU3 improved mitochondrial function in aging mice by reducing ROS-mediated apoptosis

**Fig. 4:**
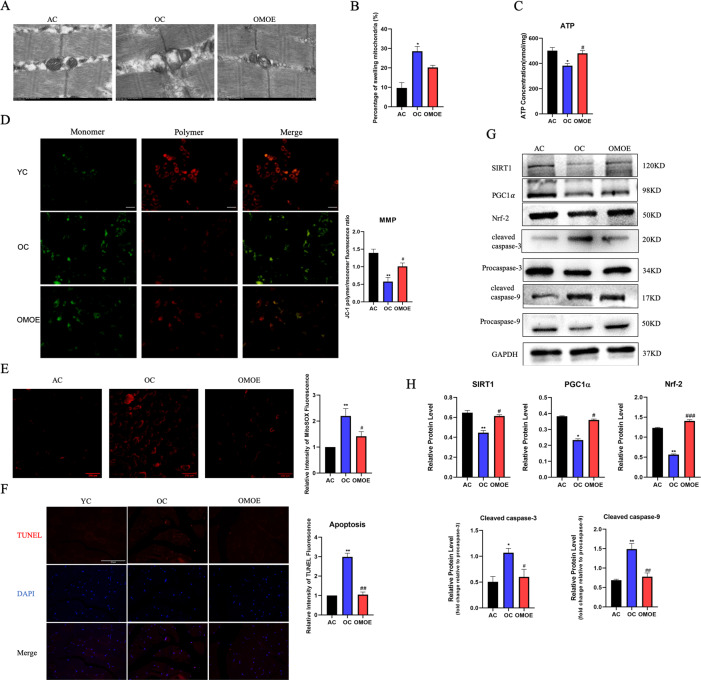
Overexpression of MICU3 improved mitochondrial function in aging mice by reducing mitoROS-mediated apoptosis. **A**, **B** Representative mitochondria were observed by transmission electron micrographs (magnification ×20,000; scale bar = 500 nm). **C** The ATP concentration in mice gastrocnemius muscle. **D** The JC-1 staining of mice primary myoblasts (scale bar = 100 μm). **E** The fluorescence staining and quantification of MitoSOX in mice gastrocnemius muscle (magnification ×200; scale bar = 100 μm). **F** The myofibers were stained with TUNEL, and the fluorescence intensity was measured by ImageJ software (magnification ×400; scale bar = 50 μm). **G**, **H** Western blot and quantification of SIRT1, PGC1*α*, Nrf-2, cleaved caspase-3, cleaved caspase-9, Procaspase-3, and Procaspase-9. GAPDH was used as the loading control. Data were expressed as means ± SEM, and data were analyzed using one-way ANOVA. *p < 0.05 vs. the AC group; ^#^*p* < 0.05 vs. the OC group; *n* = 3–4 in each group.

Mitochondrial Ca^2+^ uptake capacity was measured in isolated primary myoblasts. As shown in Fig. 3C, the uptake of mitochondrial Ca^2+^ of the OC was significantly decreased compared with the AC group. However, the forced expression of MICU3 significantly increased the mitochondrial Ca^2+^ uptake. Moreover, through TEM, the mitochondria in the OC group were swollen and showed disappearance of the cristae structure, and overexpression of MICU3 decreased the percentage of the swelling mitochondria to a certain degree. [Fig. 4A, B]. The production of ATP in the OC group was decreased while the production of ATP was increased in the OMOE group [Fig. 4C]. The mitochondrial membrane potential assay, JC-1 staining, showed that the MMP of the OC group was significantly decreased, but overexpression of MICU3 increased the MMP [Fig. 4D]. All these findings suggest that MICU3-overexpression increased mitochondrial Ca^2+^ uptake capacity, thereby improving the homeostasis and function of the mitochondria.

According to the mitoROS staining results, overexpression of MICU3 significantly reduced the level of mitoROS in aged skeletal muscle [Fig. 4E]. According to the TUNEL staining results, the forced expression of MICU3 alleviated the level of apoptosis in aging skeletal muscle [Fig. 4F]. Upregulated MICU3 decreased the expression of cleaved caspase-3 and caspase-9. Western blot results exhibited that expression of the SIRT1/PGC1 *α*/Nrf-2 pathway was decreased in the OC group [Fig. 4G, H]. However, the forced expression of MICU3 remarkably increased the expression of this pathway, indicating that MICU3 inhibited oxidative stress-related apoptosis in skeletal muscle through the SIRT1/PGC1*α*/Nrf-2 pathway.

### MICU3 alleviated the impaired differentiation capacity and the mitochondrial dysfunction in d-gal-treated C2C12 cells

**Fig. 5:**
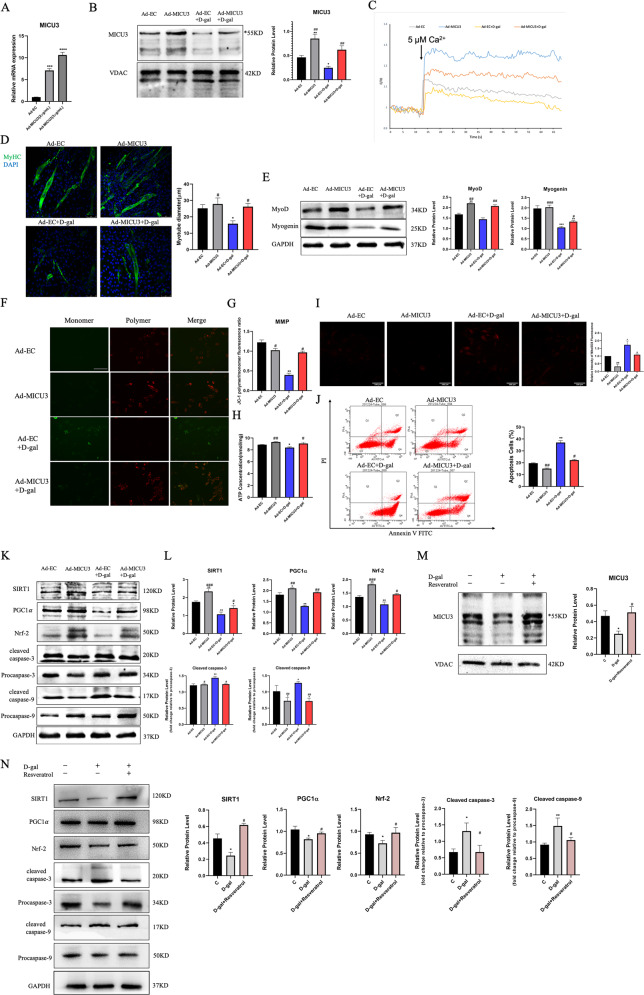
MICU3 alleviated differentiation capacity impairment and mitochondrial dysfunction in D-gal-treated C2C12 cells. **A** The mRNA level of MICU3 in C2C12 cells. **B** Western blot and quantification for MICU3 in C2C12 cells. VDAC was used as the loading control. *indicates target bands. **C** The mitochondrial calcium uptake assay in C2C12 cells. **D** The photo and quantification of myotubes (magnification = ×200; scale bar = 75 μm). **E** Western blot for MyoD and Myogenin. GAPDH was used as the loading control. **F**, **G** The JC-1 staining of C2C12 cells (scale bar = 100 μm). **H** The ATP concentration in C2C12 cells. **I** The fluorescence staining for MitoSOX (magnification = ×200; scale bar = 100 μm). **J** Representative pictures of flow cytometry analysis in Annexin V- FITC/PI staining. **K**, **L** Western blot for SIRT1, PGC1*α*, Nrf-2, cleaved caspase-3, cleaved caspase-9, Procaspase-3, and Procaspase-9. GAPDH was used as the loading control. Data were expressed as means ± SEM, and data were analyzed using one-way ANOVA and two-way ANOVA. *p < 0.05 vs. the Ad-EC group; ^#^*p* < 0.05 vs. the Ad-EC + d-gal group; *n* = 3. **M** Western blot and quantification for MICU3 in C2C12 cells treated with d-gal and resveratrol. VDAC was used as the loading control. *indicates target bands. **N** Western blot for SIRT1, PGC1*α*, Nrf-2, cleaved caspase-3, cleaved caspase-9, Procaspase-3, and Procaspase-9. GAPDH was used as the loading control. Data were expressed as means ± SEM, and data were analyzed using one-way ANOVA. *p < 0.05 vs. The C (d-gal = 0 g/L) group; ^#^*p* < 0.05 vs. the d-gal (d-gal = 20 g/L) group; *n* = 3.

Our previous study demonstrated that d-gal induced C2C12 cell senescence and the optimal concentration of d-gal was 20 g/L. Our data indicate that d-gal induced the mitochondrial Ca^2+^-related dysfunction and impaired the differentiation capacity. Since MICU3 was identified as being decreased due to d-gal, we next determined whether overexpression of MICU3 played an important role in anti-apoptosis in d-gal-treated C2C12 cells. The PCR and western blot data revealed that expression of MICU3 increased significantly after plasmid transfection [Fig. 5A, B]. As shown in Fig. 5D, the size of myotubes in the d-gal-treated groups was smaller than in the other groups, but was alleviated with overexpression of MICU3. Similarly, western blot results showed that the myogenic markers (MyoD and myogenin) were downregulated by d-gal but upregulated by forced expression of MICU3 [Fig. 5E].

As shown in Fig. 5C, the mitochondrial Ca^2+^ uptake decreased significantly after d-gal treatment, but could be alleviated to a certain degree by overexpression of MICU3. The JC-1 staining results demonstrated that d-gal treatment was characterized by significantly higher JC-1 polymer fluorescence and reduced JC-1 monomer fluorescence. However, upregulation of MICU3 decreased the JC-1 polymer fluorescence and increased the monomer fluorescence [Fig. 5F, G]. Similarly, ATP production decreased after d-gal treatment but increased with overexpression of MICU3 [Fig. 5H]. The above results indicate that MICU3 could alleviate mitochondrial function disorder induced by d-gal.

Additionally, we found that d-gal treatment increased the production of mitoROS, which could be reduced by the overexpression of MICU3 [Fig. 5I]. The apoptosis assay revealed that apoptosis was increased after d-gal treatment but decreased after forced expression of MICU3 [Fig. 5J]. Furthermore, the western blot results showed that the SIRT1/PGC1*α*/Nrf-2 pathway was decreased with cleaved caspase-3 and cleaved caspase-9 increasing after d-gal treatment, but MICU3 reduced the cleavage of caspase-3 and caspase-9 [Fig. 5K, L]. In addition, resveratrol, a classic antioxidant, could produce a similar antioxidant and anti-apoptosis effect as overexpression of MICU3 [Fig. 5M, N], indicating that the effect of MICU3 may be related to oxidative stress.

### MICU3-mediated mitochondrial Ca^2+^ enhanced the antioxidant effect

**Fig. 6:**
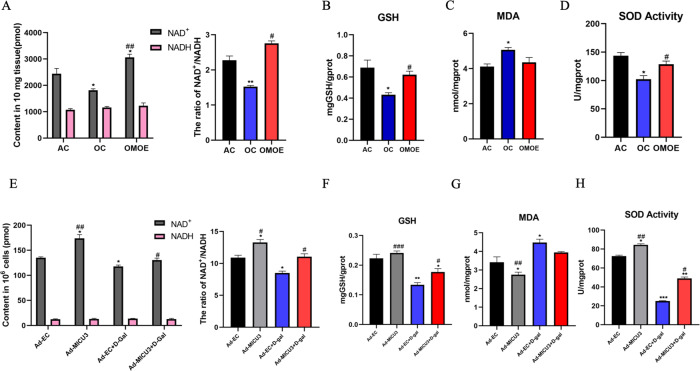
MICU3-mediated mitochondrial Ca^2+^-enhanced antioxidant effect. **A** NAD^+^, NADH contents, and the ratio of NAD^+^ and NADH in mice gastrocnemius muscle. **B** The GSH contents in mice gastrocnemius muscle. **C** The MDA contents in mice gastrocnemius muscle. **D** The activity of SOD in mice gastrocnemius muscle. *p < 0.05 vs. the AC group; ^#^*p* < 0.05 vs. the OC group; *n* = 3 mice in each group. **E** NAD^+^, NADH contents, and the ratio of NAD^+^ and NADH in C2C12 cells. **F** The GSH contents in C2C12 cells. **G** The MDA contents in C2C12 cells. **H** The activity of SOD in C2C12 cells. *p < 0.05 vs. the Ad-EC group; ^#^*p* < 0.05 vs. the Ad-EC + d-gal group; *n* = 3. Data were expressed as means ± SEM, and data were analyzed using a one-way ANOVA.

Since mitochondrial Ca^2+^ is essential to the TCA cycle, which is essential to the cellular antioxidant system [32, 33], it was reasonable to hypothesize that MICU3-mediated mitochondrial Ca^2+^ would enhance the antioxidant system. Our in vivo experimental data showed that aging reduced NAD^+^/NADH and glutathione (GSH) production and the activity of SOD, with an increase in malondialdehyde (MDA). However, MICU3 increased the regeneration of NAD^+^, GSH, and the activity of SOD without affecting the MDA concentration [Fig. 6A–D]. Similarly, we found that in in vitro experiments, the d-gal decreased NAD^+^/NADH and GSH production and the activity of SOD, and increased MDA, while MICU3-overexpression had the opposite effect [Fig. 6E–H]. Collectively, these findings suggest that MICU3-overexpression and subsequent elevation of mitochondrial Ca^2+^ enhanced the antioxidant system.

### SIRT1 played a critical role in the effect of MICU3

**Fig. 7:**
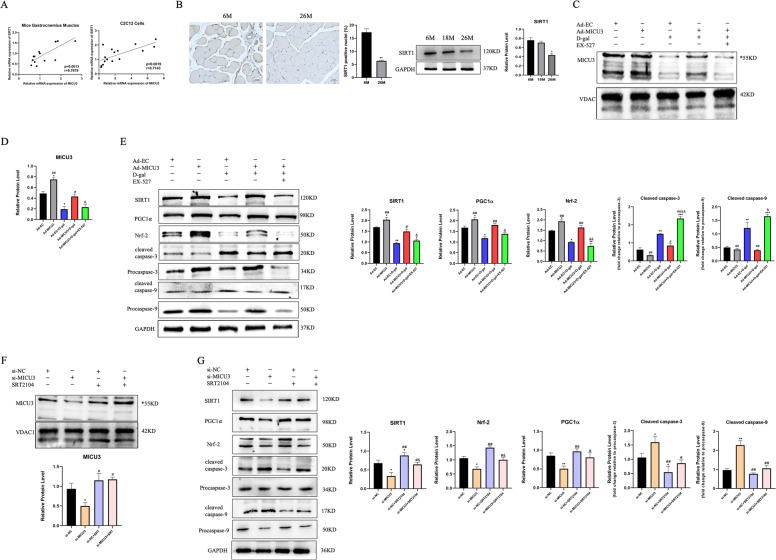
SIRT1 plays a critical role in the effect of MICU3. **A** Correlation graphs between the mRNA expression of MICU3 and SIRT1. B IHC and western blot for SIRT1 in mice from different age groups (magnification ×400; scale bar = 20 μm, *p < 0.05 vs. the 6M group; *n* = 3). **C**, **D** Western blot and quantification of MICU3. VDAC was used as the loading control. *indicates target bands. **E** Western blot and quantification of SIRT1, PGC1*α*, Nrf-2, cleaved caspase-3, cleaved caspase-9, Procaspase-3, and Procaspase-9. GAPDH was used as the loading control. (*p < 0.05 vs. the Ad-EC group; ^#^*p* < 0.05 vs. the Ad-EC + d-gal group; ^&^*p* < 0.05 vs. the Ad-MICU3 + d-gal group; *n* = 3). **F** Western blot and quantification of MICU3. VDAC was used as the loading control. *indicates target bands. **G** Western blot and quantification of SIRT1, PGC1*α*, Nrf-2, cleaved caspase-3, cleaved caspase-9, Procaspase-3, and Procaspase-9. GAPDH was used as the loading control. (*p < 0.05 vs. the si-NC group; ^#^*p* < 0.05 vs. the si- MICU3 group; ^&^*p* < 0.05 vs. the si-NC + SRT2104 group; *n* = 3). Data were expressed as means ± SEM, and data were analyzed using a one-way ANOVA.

SIRT1 is a downstream effector of NAD^+^ and plays an essential role in aging, apoptosis, and oxidative stress [34–36]. Our data show that SIRT1 might correlate with MICU3. Pearson correlation analysis indicates a significant positive correlation between mRNA expression levels of SIRT1 and MICU3 in both muscle tissues of aging mice and senescent C2C12 cells [Fig. 7A]. Besides, IHC and western blot results demonstrated that SIRT1 was also downregulated in the skeletal muscle of aging mice [Fig. 7B]. Thus, we utilized EX-527, a selective SIRT1 inhibitor, to suppress SIRT1. After inhibiting SIRT1, the downstream effectors PGC1*α* and Nrf-2, were inhibited. In addition, the anti-apoptosis effect of MICU3 was blocked by the inhibition of SIRT1 [Fig. 7E]. Next, we applied SRT2104, a specific SIRT1 activator, to activate SIRT1 [37]. We found the expression of downstream PGC1*α* and Nrf-2 was increased by activation of SIRT1, with reduced cleavage of caspase-3 and caspase-9 [Fig. 7G]. Furthermore, the expression of MICU3 was decreased by the inhibition of SIRT1, and increased by the activation of SIRT1, indicating that SIRT1 may affect MICU3 expression in turn [Fig. 7C, D, F]. Together, these results suggest that MICU3 promotes mitochondrial Ca^2+^ homeostasis and the antioxidant response partially through the SIRT1/PGC1*α*/Nrf-2 pathway, and that SIRT1 might be involved in the downregulation of MICU3 in the skeletal muscle of aging mice.

## Discussion

Aging is a process that involves multiple factors and organs. Loss of muscle strength exerts a considerable impact on the quality of life and mortality of older adults. However, molecular pathologic mechanisms of muscle aging are still unclear. Here, we have made several important observations. First, we validated that MICU3 was downregulated in the skeletal muscle of aging mice. Second, overexpression of MICU3 protected the skeletal muscle mass and function. Third, the anti-apoptotic effect of MICU3 was mediated by promoting mitochondrial Ca^2+^ uptake and subsequently activating the antioxidant system. Last, SIRT1 played a critical role in the effect of MICU3. Collectively, our study has established a novel mechanism and showed that impaired MICU3 signaling contributed to skeletal muscle aging.

Changes in the morphology and content of human skeletal muscle mitochondria are associated with age-related loss of muscle mass and function [38]. Mitochondrial Ca^2+^ plays a role in regulating the intrinsic functions of mitochondria, and one study has reported that MCU-dependent mitochondrial Ca^2+^ uptake has a trophic effect [26]. Another study found that mutations in the MICU1 gene caused skeletal muscle fatigue by affecting mitochondrial calcium signaling [21]. However, the effect of MICU3 in skeletal muscle has not been previously investigated.

MICU3 is an EF-hand-containing protein that is resident in the mitochondrial intermembrane space and has been proposed to be an enhancer of MCU-mediated mitochondrial Ca^2+^ uptake [39, 40]. In this study, we demonstrated that upregulation of MICU3 increased the mitochondrial Ca^2+^ uptake in primary myoblasts and C2C12 cells during high [Ca^2+^]_cyto_ pulse. As indicated by previous evidence, skeletal muscle mitochondria exposed to high [Ca^2+^]_cyto_ rapidly sequesters Ca^2+^ during excitation-contraction coupling. Therefore, it is reasonable that the restoration of MICU3 has an activating effect on mitochondrial Ca^2+^ uptake in aging skeletal muscle.

Mitochondrial Ca^2+^ overload is detrimental and could cause the opening of the permeability transition pore (PTP), mitochondria swelling, and ROS production [41]. However, compared with these conditions, the effect of elevated mitochondrial Ca^2+^ might be different in the aging skeletal muscle. In our study, the mitochondrial Ca^2+^ uptake capacity was reduced in the skeletal muscle of aging mice. Therefore, the recovery of mitochondrial Ca^2+^ uptake promoted skeletal muscle function in the aged mice. Our data showed that MICU3-overexpression recovered the skeletal muscle physical function and mass, suggesting that impaired mitochondrial Ca^2+^ handling induced by downregulated MICU3 could be a cause of the dysfunction in aging mice. However, other factors besides defects in MICU3 could be responsible for the impaired function of aging skeletal muscle, which could be alleviated, to a certain degree, by MICU3-mediated mitochondrial Ca^2+^ signaling.

Age-associated diseases are characterized by the progressive loss of organ function due to the accumulation of ROS-induced damage to biomolecules since the ability to counteract the continuous and large generation of ROS becomes increasingly inefficient with aging. This positions mitochondrial dysfunction as a central pathogenic mechanism for both pathological conditions [42]. ROS generation plays a role in the age-related loss of muscle mass and function [43]. And one study has drawn an association between mitochondrial Ca^2+^ uptake and mitochondrial ROS production [44]. Here, we demonstrated that overexpression of MICU3 reduced skeletal muscle apoptosis by reducing the mitochondrial ROS level. In addition, we found that MICU3-mediated mitochondrial Ca^2+^ uptake inhibited mitochondrial ROS-triggered apoptosis through the SIRT1/PGC1*α*/Nrf-2 pathway. In contrast, the overexpression of MICU3 in skeletal muscle did not benefit young and healthy mice but induced much lower skeletal muscle function (data not shown). One possible explanation might be that overexpression of MICU3 induced mitochondrial Ca^2+^ overload and subsequently caused skeletal muscle apoptosis. One study reported that sarcoplasmic reticulum Ca^2+^ leak-mediated mitochondrial Ca^2+^ accumulation could trigger mitochondrial dysfunction and increase the production of ROS [45]. The above results indicate the critical role of mitochondrial Ca^2+^ homeostasis in maintaining skeletal muscle function. Particularly, oxidative stress damage induced by Ca^2+^ overload should be taken care of during MICU3-based treatment for muscle aging.

We have further found that the effects of MICU3 in muscle aging were partly due to SIRT1. SIRT1 is a NAD^+^ sensitive deacetylase that is associated with lifespan and is believed to have beneficial effects on counter aging [46]. In addition to ameliorating insulin assistance, SIRT1 has also been known to promote the activation of the Nrf-2/ARE pathway and improve oxidative stress in diabetic nephropathy [47]. Increasing the total level and nuclear activity of SIRT1 could reduce the expression of caspase-3 and Bax and increase SOD and GSH levels in doxorubicin-induced nephropathy [48]. SRT2104 confers further protection against mitoROS by increasing SIRT1 levels and improving antioxidant production, which reduces mitochondrial-associated apoptotic signaling and cell death in myoblasts [49]. In our study, inhibiting SIRT1 with Ex-527 reversed the effect of MICU3, and activating SIRT1 with SRT2104 improved the oxidative stress and apoptosis induced by the deficiency of MICU3. Moreover, the expression of MICU3 was positively correlated with SIRT1, which provided a clue for understanding the regulatory mechanisms of MICU3 expression in the skeletal muscle of aging mice.

There are several limitations to our study. First, not all the conclusions were derived from in vivo studies. Second, the causes of downregulated MICU3 in skeletal muscle of aging mice remain largely unknown, although we have found a possible involvement of SIRT1. Third, the mitochondrial Ca^2+^ uptake only tested primary myoblasts from three mice per group. Despite these limitations, we believe that our study provides new insights into skeletal muscle aging.

In summary, our study provides evidence that the downregulation of MICU3 promotes skeletal muscle weakness during aging, and that the reconstitution of MICU3 alleviates skeletal muscle function and mass via a mitochondrial Ca^2+^-dependent antioxidant pathway. This suggests a potential therapeutic target to mitigate skeletal muscle function in aging.

## Materials and methods

### Animals and treatment

All male C57BL/6J mice were purchased from the Experimental Animal Center of Chongqing Medical University. All animal experiments were carried out in accordance with the Guide for the Care and Use of Laboratory Animals of the National Institutes of Health. The animals were divided into three groups as follows: (1) adult control group (AC group, 4 months old at the beginning of treatment, male, *n* = 12); (2) old control group (OC group, 24 months old at the beginning of treatment, male, *n* = 12); and (3) old MICU3-overexpression group (OMOE group, 24 months old at the beginning of treatment, male, *n* = 12). Recombinant AAV9 overexpression mouse MICU3 (NM_030110) and AAV9-GV388 vehicle were constructed by Genechem Co., Ltd. For AAV9 administration, the hindlimbs of the mice were shaved. AAV9 (1 × 10^12^ vg/mL) was injected into the bilateral gastrocnemius muscles (3 sites/side, 6 μL/site) of the mice. The mice in the OMOE group had an intramuscular injection of recombinant AAV9 overexpression mouse MICU3. The mice in the AC group and OC group received an intramuscular injection of an AAV9 vehicle. The transfection efficiency was evaluated by real-time PCR and western blot analysis eight weeks after AAV9 injection.

### Isolation of primary myoblasts

Primary myoblasts were isolated from the gastrocnemius muscles of mice that have been injected with AAV9. Myoblasts were isolated by dissociation with 0.05% Trypsin/EDTA (Servicebio, Wuhan, China) and 5 mg/mL collagenase type IV (Worthington, USA) in DMEM at 37 °C for 30 min. The mixture was then filtered through a 70 μm cell strainer (Corning, USA). Cells were cultured in DMEM containing 20% FBS (Gibco, USA) and 1% penicillin/streptomycin (Gibco, USA) at 37 °C and 5% CO_2_. After 30 min the medium was collected and pre-plated for a second time. After a further 30 min, the medium was transferred to a flask coated with Poly-D-Lysin (PDL, Sigma, USA). At ~70–80% confluence medium was replaced with a differentiation medium made up of DMEM containing 2% Horse serum and 1% penicillin/streptomycin.

### Cell culture and treatment

C2C12 cells were obtained from ATCC and used within the first 15 passages. C2C12 cells were cultured in proliferation medium (DMEM + 10% FBS + 1% penicillin/streptomycin [P/S]) with 5% CO_2_ and 95% air. To gain insight into the effect of MICU3 on C2C12 cells, small interfering RNA (siRNA, GeneCopoeia) was used to knock down intracellular MICU3, and overexpression plasmid (EX-Mm-25481-m39, GeneCopoeia) was used to overexpress MICU3. C2C12 cells were seeded into 6-well plates and cultured to 50% confluency. siRNA and overexpression plasmid were transfected into cells at a concentration of 50 nM. The sequences of siRNAs are as follows: (1) siRNA-NC sense: UUCUCCGAACGUGUCACGUdTdT; antisense: ACGUGACACGUUCGGAGAAdTdT; (2) siRNA-MICU3 sense 1: UCAUAAUUCCAAUAAAUUCUU; antisense 1: GAAUUUAUUGGAAUUAUGAAA; siRNA-MICU3 sense 2: GAGAGACGGUUUCGUUUAU; antisense 2: AUAAACGAAACCGUCUCUC; siRNA-MICU3 sense 3: UAUCCAUAAAGUUGAAGACGC; antisense 3: GUCUUCAACUUUAUGGAUACC. EndofectinTM-Max (GeneCopoeia) was used to transfer siRNAs, overexpression plasmid, and empty control plasmid according to the operation manual. After 8 h, the medium was changed to a differentiation medium (DMEM + 2% horse serum + 1% P/S) to induce differentiation. The differentiation medium was changed at least every 2 days. To test the effect of d-gal on C2C12 cells, cells were incubated with different doses of d-gal (0, 20, or 40 g/L, Sigma, USA). To test the effect of antioxidants on senescent cells, cells were treated with 50 μmol/L resveratrol (MedChemExpress, USA). To probe the role of SIRT1, cells were treated with 3 μmol/L SRT2104 (Beyotime, China), a specific SIRT1 activator, or 20 μmol/L EX-527 (Beyotime, China), a SIRT1 selective inhibitor. All groups were given equal amounts of solvent to create a uniform condition.

### Measurement of myotube diameter

After treatment, cells were induced differentiation for at least 8 days. Myotubes were fixed 4% formaldehyde for 15 min, incubated with 0.5% Triton X-100 for 15 min, and then blocked with 4% BSA for 1 h. Then the myotubes were incubated with MyHC primary antibody (1:200) at 4 °C overnight. After washing with PBS for three times, myotubes were incubated with secondary antibody (Alex Fluor 488-conjugated, ThermoFisher) and DAPI for 1 h at room temperature. The myotubes were photographed with a confocal laser scanning microscope (SP8 WLL, Leica, Germany). And the average diameters were measured by ImageJ software. Ten random fields were photographed for each sample.

### SA-*β*-gal staining

Senescence-associated *β*-galactosidase (SA-*β*-gal) staining was performed using a SA-*β*-gal staining kit (CST, USA) according to the manufacturer’s protocol. C2C12 cells were seeded on 6-well plates and treated with different doses of d-gal for 48 h, then cells were stained with the SA-*β*-gal and cultured in a 37 °C dry incubator (no CO_2_ condition). The SA-*β*-gal-positive cells exhibited a blue color under a phase-contrast microscope. The percentages of the positive area were measured by ImageJ software. Each experiment was repeated three times.

### Exhausted exercise

To assess the muscle function of the animals, treadmill (Zhongshi, China) running to exhaustion was performed using the following protocol [50]: (1) 5 m/min for 5 min; (2) 10 m/min for 5 min; (3) 15 m/min for 5 min; (4) kept 20 m/min until the animals were exhausted. The furthest running distance readings were recorded.

### Dual-energy X-ray absorptiometry (DEXA)

All mice were anesthetized by 4% Chloral hydrate (0.1 mL/10 g). Body composition was assessed using dual-energy X-ray absorptiometry (DEXA, Hologic Discovery A [Hologic Inc., USA]).

### Tissue preparation

After the mice had been killed, the gastrocnemius muscles, the tibialis anterior muscle, and myocardium from all mice were harvested and placed into 1.5 mL cryotubes, frozen in liquid nitrogen, and stored at −80 °C for real-time polymerase chain reaction (RT-PCR) and western blot analysis. For hematoxylin and eosin (H&E), tissue fluorescence detection, and immunohistochemistry, the gastrocnemius muscles were removed and fixed in 4% paraformaldehyde at 4 °C for 24 h. For transmission electron microscopy, the gastrocnemius muscles were removed and fixed for 2 h in 2.5% glutaraldehyde at room temperature and then stored at 4 °C for 24 h. For mitochondria analysis, the gastrocnemius muscles were removed and placed on ice, and the mitochondria were extracted within one hour.

### Staining and IHC

The gastrocnemius muscles were embedded in paraffin, and tissue sections (5 μm) were obtained. The hematoxylin and eosin (H&E) staining was performed using a hematoxylin and eosin stain kit (Wanleibio, China). For IHC analyses, deparaffinized and hydrated sections were incubated in 1 mM citrate buffer (pH 6.0) at 95 °C for 30 min in a microwave oven for heat-induced epitope retrieval. Then, the sections were incubated with 3% H_2_O_2_ for 20 min. Nonspecific binding was blocked via incubation with 10% goat serum for 30 min at 37 °C. Then, the sections were treated with primary antibodies against MICU3 and SIRT1 overnight at 4 °C. After washing with PBS, the sections were incubated with a biotinylated secondary antibody (Zhongshan Inc. Beijing, China) for 30 min at 37 °C. Finally, immunoreactivity was detected by diaminobenzidine. The sections were dehydrated and mounted on slides. All sections were examined blindly using a light microscope (ZEISS, Germany) at ×200, ×400 magnification. The images were analyzed using the ImageJ software.

### Immunofluorescence

The gastrocnemius muscles were embedded in NEG50 gum (ThermoFisher, USA), and stored at −80 °C until use. Cryosections of the muscle were mounted on a slide and then horizontally sectioned into 10-μm slices. Mounted sections were blocked in blocking buffer (10% goat serum in PBS) for 1 h at room temperature. Then, the slides were incubated overnight at 4 °C with a primary antibody against Desmin (1:200). The slides were incubated with a secondary antibody (Alex Fluor cy3-conjugated, ThermoFisher) for 1 h at room temperature, and DAPI (Life Technologies, USA) was used to stain the nuclei. The sections were viewed and photomicrographs were captured under a confocal laser scanning microscope (SP8 WLL, Leica, Germany).

### Detection of mitochondrial reactive oxygen species (mitoROS)

MitoROS was detected by the fluorescent probe MitoSOX (Invitrogen) according to the manufacturer’s protocols. For C2C12 cells, images were captured by laser confocal microscope (SP8 WLL, Leica, Germany). For muscle section, images were captured under a fluorescence microscope (Olympus BX53, Tokyo, Japan). The intensity of fluorescence was analyzed using ImageJ software.

### Transmission electron microscopy

Gastrocnemius muscle tissue was fixed overnight in 2.5% glutaraldehyde fixative at 4 °C and then randomly cut into five to six tissue blocks (1 mm^3^) in the same area between groups. All samples were subsequently processed for transmission electron microscopy (TEM, Hitachi, Japan) at the Institute of Life Science of Chongqing Medical University. From each section, ten fields of images were randomly captured at a magnification of ×20,000.

### Tissue mitochondria isolation

The tissue mitochondria isolation was performed using a Tissue Mitochondrial Extraction Kit (Beyotime, China) according to the manufacturer’s protocol. The concentration of mitochondria was determined by the bicinchoninic acid (BCA) method (Beyotime, China). The isolated mitochondria were used for tests within 1 h.

### Measurement of mitochondrial Ca^2+^ uptake

The fluorescent dye Rhod-2/AM (Yeasen, China) and Mito-Tracker Green (Beyotime, China) were used to measure primary myoblasts and C2C12 cells mitochondrial Ca^2+^ uptake, following the manufacturer’s instructions [51]. Briefly, primary myoblasts or C2C12 cells were incubated with Rhod-2/AM and Mito-Tracker Green to allow the cells to load the dyes according to the protocol. Then, the cells were permeabilized by 0.005% saponin (MedChemExpress, USA) for ~1 min, and the permeabilization solution was replaced with Ca^2+^-free HBSS solution. Cells were viewed with a spinning disk confocal super-resolution microscope (SpinSR10, Olympus, Tokyo, Japan) at the following wavelengths: 549 nm (excitation) and 578 nm (emission), and the frame rate was 2 frames/s. At the 15 s point, the Ca^2+^-free HBSS solution was replaced with 5 μmol/L CaCl_2_ solution and continued to acquire images for an extra 50 s. Select the colocalization regions of Rhod-2/AM and Mito-Tracker Green to calculate the intensity of Rhod-2/AM by using Imaris software (Oxford, England) and ImageJ software. All the experiments were repeated three times.

### RNA extraction and RT-PCR

RNA isolation, cDNA synthesis, and RT-PCR were performed using RNAiso Reagent, PrimeScript^TM^ RT Reagent Kit with gDNA Eraser, and SYBR Green PCR Master Mix (TaKaRa, Japan), respectively. RT-PCR was performed using the CFX96^TM^ Real-Time System (Bio-Rad, USA) under the following conditions: denaturation at 95 °C for 2 min; 40 cycles of amplification at 95 °C for 30 s, 60 °C for 30 s, and 72 °C for 30 s; and a final extension at 72 °C for 3 min. The specific mouse primers used in this study are listed in Table 1. The gene expression data were calculated by the 2^−△△Ct^ method and normalized to GAPDH. All experiments were performed in triplicate.

**Table 1:** Oligonucleotide primer sequences used in this study.

Gene name	Sequence 5′–3′ (forward)	Sequence 5′–3′ (reverse)
MICU3	CAGATGCTGGGGAACTTGTCT	TGTCATCAGCACGCTCTGC
EMRE	CCATTGTGATCCCCTTTCTCTA	TGGGACAAAAATGTCATGTTCC
SIRT1	AGGGAACCTTTGCCTCATCTAC	GGTGGCAACTCTGATAAATGAAC
GAPDH	GACATCAAGAAGGTGGTGAAGC	GAAGGTGGAAGAGTGGGAGTT

### Western blot

Gastrocnemius muscles and C2C12 cells were lysed with ice-cold RIPA buffer (Beyotime, China) containing a protease and phosphatase inhibitor cocktail (Roche, Switzerland). The protein concentration was determined using the BCA Kit. Equal amounts of protein (35 μg) were separated by 10% SDS-polyacrylamide gels and then transferred onto polyvinylidene difluoride (PVDF) membranes (Millipore, USA). The membranes were then incubated with primary antibodies at 4 °C overnight. All the primary antibodies used in this study are listed in Table 2. Then, the membranes were incubated with the corresponding goat anti-mouse (1:8000, Proteintech, China) or anti-rabbit (1:8000, Proteintech, China) secondary antibody at room temperature for 1 h. The membranes were subsequently washed three times with TBST and the reactive proteins were detected by an enhanced chemiluminescence reagent (Millipore, USA). The bands’ grayscale densities were analyzed by the Fusion imaging system (Fusion Imaging Software, USA).

**Table 2:** Primary antibodies used for western blotting and immunohistochemistry.

Antibody	Company (Lot.)	Working dilutions
MICU3	Sigma (R11856)	WB:1/250 IHC:1/150
SIRT1	Abcam (ab32441)	WB:1/500 IHC:1/100
Desmin	Wanleibio (WL0174)	IF:1/200
MCU	Proteintech (26312-1-AP)	WB:1/2000
VDAC	Wanleibio (WL02790)	WB:1/500
PGC1α	Abcam (ab106814)	WB:1/500
Nrf-2	ZENBIO (340675)	WB:1/1000
GAPDH	Proteintech (60004-1-lg)	WB:1/5000
MyoD	Santa Cruz (sc-71629)	WB:1/200
Myogenin	Santa Cruz (sc-52903)	WB:1/200
Cleaved Caspase-3	Wanleibio (WL02117)	WB:1/500
Cleaved Caspase-9	Wanleibio (WL01838)	WB:1/500
Caspase-9	Wanleibio (WL03421)	WB:1/500
Caspase-3	Wanleibio (WL04004)	WB:1/500
P16INK	Wanleibio (WLH3673)	WB:1/500
P53	Abcam (ab26)	WB:1/1000
MyHC	Santa Cruz (sc-376157)	WB: 1/1000 IF: 1/200

### ATP assay

ATP concentration measurement was carried out in accordance with the protocol of the ATP Assay Kit (Beyotime, China). The luminescence was measured by the Tecan Infinite 200Pro (Tecan, Austria). The protein concentration was determined by the BCA method. The ATP concentration was standardized by protein concentration.

### Mitochondrial membrane potential measurement

According to the JC-1 Assay Kit (Yeasen Biotechnology, China) protocol, the cultured C2C12 cells and primary myoblasts were washed with PBS and incubated with a differentiation medium containing JC-1 probe for 20 min at 37 °C in the dark. After washing two times with ice-cold buffer, pictures were taken of the cells with an Olympus BX51 microscope (Tokyo, Japan) at ×200 magnification. JC-1 level, the indicator of mitochondrial membrane potential, was expressed by the ratio of polymer to monomer fluorescence intensities.

### Apoptosis measurement

For in vitro experiments, C2C12 cells were harvested in Annexin V binding buffer and incubated in Annexin V-FITC and PI for 15 min at room temperature in the dark. The cell suspension was then diluted with Annexin V binding buffer and measured by flow cytometry. For in vivo experiments, the muscles were embedded in paraffin and cut into sections (5 μm). The sections were stained with the Fluorescence and Colorimetric TUNEL Apoptosis Assay Kit (Servicebio, China) according to the protocol. All sections were examined using a confocal laser scanning microscope (ZEISS LSM800, Germany) at ×200, ×400 magnification. The images were analyzed using the ImageJ software.

### Detection of NAD^+^/NADH, GSH, MDA contents, and activity of SOD

The concentration of NAD^+^/NADH was measured by the NAD^+^/NADH assay kit (Beyotime, China) according to the manufacturer’s protocol. Contents of GSH (Glutathione) and MDA (Malondialdehyde) were measured by the GSH assay kit and MDA assay kit (Jiancheng Biotechnology, Nanjing, China), respectively, according to the manufacturers’ instructions. The activity of SOD was measured by the SOD assay kit (Jiancheng Biotechnology, Nanjing, China).

### Statistical analysis

All data are presented as the mean ± standard error of the mean (SEM) and analyzed with GraphPad Prism 8.0 software. All comparisons were performed with the Student’s *t*-test for comparing two groups, one-way ANOVA for three groups or more groups. Pearson’s correlation coefficient was used to evaluate the correlation between MICU3 and SIRT1 expression. A value of *p* < 0.05 was considered statistically significant.

## Supplementary information

authors’ statement

reproducibility checklist

## Data Availability

All data used and analyzed in this study are available from the corresponding author.
