# Supplementary material for: MICU3 regulates mitochondrial Ca2+-dependent antioxidant response in skeletal muscle aging
Source: Cell Death Dis. 2021 Nov 29;12(12):1115. doi: 10.1038/s41419-021-04400-5 (PMC8630021; doi:10.1038/s41419-021-04400-5)
Supplement: Supplementary file 1 — authors’ statement [file 41419_2021_4400_MOESM1_ESM.docx]

Dear co-authors，

  It is so important to inform you that I have added Jing Zhou as a co-author of our manuscript entitled "MICU3 regulates mitochondrial Ca2+-dependent antioxidant response in skeletal muscle aging" because of her contribution to cell experiments and data analysis.

The journal of Cell Death & Disease asks all authors to confirming that you agree to this change.

If you have any questions about this change, please contact me.

Sincerely

Xiaoqian

*Yun-Fei Yang (*[*1368425450@qq.com*](mailto:1368425450@qq.com)*):*

Dear professor Xiao,

I acknowledge Miss Zhou's contribution to this manuscript and agree to the change.

Yours,

Yangyunfei

*Wu Yang (1107080687@qq.com):*

I agree to add her as a co-author.

*Ai Guo (976847223@qq.com):*

I agree to this change.

*Yong-Xin Wu (724346021@qq.com):*

Dear Prof. Xiao

I agree with the change without any questions.

Sincerely

Wu Yongxin

*Jing Zhou (zj4321228@sina.com):*

I agree to be co-author of this submission.

Regards,

Jing Zhou

*Zhi-yin Liao (136920061@qq.com):*

I agree add Jing Zhou as a co-author of the submission.

Regards,

Zhiyin Liao

*Zhen Fan (534384186@qq.com):*

I agree Jing Zhou to be co-author of this submission.

Regards

Zhen Fan

*Qiu-Nan Chen (280021713@qq.com):*

I agree that Jing Zhou to be co-author of this submission.

Regards,

Qiunan,Chen

*Jing Yu (yuxiaojing1015@126.com):*

Dear Dr. Xiao
I agree Dr. Zhou to be the co-author in this manuscript.
Best regard,
Jing Yu

*Jiang-Hao Wu (767519964@qq.com):*

I agree with this change without objection.

Regards

Jianghao Wu
